# Resistance of External Thermal Insulation Systems with Fire Barriers to Long-Lasting Weathering

**DOI:** 10.3390/ma17133113

**Published:** 2024-06-25

**Authors:** Ewa Sudoł, Artur Piekarczuk, Ewelina Kozikowska, Aleksandra Mazurek

**Affiliations:** 1 Construction Materials Engineering Department, Instytut Techniki Budowlanej, 00-611 Warsaw, Poland; e.kozikowska@itb.pl; 2 Building Structures, Geotechnics and Concrete Department, Instytut Techniki Budowlanej, 00-611 Warsaw, Poland; a.piekarczuk@itb.pl (A.P.); a.mazurek@itb.pl (A.M.)

**Keywords:** ETICS, hydrothermal cycles, watertightness, performance, fire barriers, 3D scanning

## Abstract

Fire barriers are used to reduce the risk of fire spreading over façades. External thermal insulation composite systems consist of mineral wool strips embedded in a layer of another thermal insulation material. A system configured in this manner, beyond standard solutions, can be more susceptible to environmental factors. In this study, an expanded polystyrene-based system with a mineral wool fire barrier was subjected to weathering conditions. In view of climate change, nonconventional long-lasting exposure simulating the effects of intensive atmospheric factors was applied. Two exposure sequences were used, each covering 80 cycles of heating and wetting, five cycles of heating and cooling, and 30 cycles of wetting, freezing, and thawing. Significant changes were observed in the first sequence. The second sequence caused rendering system cracks wider than 0.2 mm. This indicated a loss of watertightness. A new approach of 3D scanning with inspection analysis was used to assess the deformations. It showed deformation amounted to 0.7 mm within the MW strip. The methods used previously did not allow this level of deformation to be recorded.

## 1. Introduction

For over 60 years, external thermal insulation composite systems (ETICS) have been among the most popular methods for improving the energy efficiency of buildings and renewing facades [1]. It is estimated that ca. 215–230 million m^2^ of building walls are thermally insulated in this manner [2]. ETICS cover a thermal insulation material fixed to the substrate and a finishing layer made on-site with no air voids or intermediate layers. A rendering system consists of a reinforcement layer composed of an adhesive mortar with embedded fibreglass mesh and a topcoat covering a primer (optionally), thin-coat render, and, in some systems, a paint coat and impregnate [3,4]. In practice, various thermal insulation materials are used in the ETICS. Typically, these are expanded polystyrene (EPS) or mineral wool (MW) [5,6]. However, according to the European Assessment Document EAD 040083-00-0404 [7], using various thermal insulation materials is not acceptable in one ETICS. None of the several hundred European Technical Assessments (ETA) issued so far for ETICS cover thermal insulation systems that combine various thermal insulation materials [3]. Nevertheless, many countries have introduced the mandatory use of fire barriers to reduce the risk of fire spreading between floors over the facade [8,9,10]. The requirement for ETICS applies especially to EPS-based systems, which are typically classified as self-extinguishing flammable materials [11]. Strips of non-flammable MW were introduced into the EPS layer [8]. MW strips are placed between the storeys, at the floor levels, and, in some cases, above the windows and doors [9]. The first strip should be made immediately above the plinth, another one at the floor level above the first story on the ground, and the following ones at successive floor levels, above selected stories, with or without strips above windows and doors, depending on the arrangement of the windows, roofing type, thermal insulation layer thickness, and building height [8].

The requirement to use fire barriers applies to thermal insulation systems that are purely bonded, bonded with supplementary mechanical fixings, mechanically fixed with supplementary adhesive, and purely mechanically fixed, regardless of the building type and height [8,9]. Moreover, the strips between the storeys are assumed to be at least 200 mm high, with a thickness corresponding to the EPS thickness. They should be made from MW, according to EN 13162 [12], with A1 or A2-s,d0 fire rating according to EN 13501-1 [13]. MW strips should be fixed to the substrate with cement-based adhesive applied all over the surface and dowels (steel stem and plastic plate) with a diameter of at least 60 mm. The dowels are arranged within the strip’s horizontal axis, no farther than 150 mm from the board’s vertical edge or 450 mm from one another [8].

Guidelines on the use of fire barriers in ETICS were developed considering the fire safety of buildings. The aspect affecting the building structure’s fulfilment of other basic requirements [14] was skipped, including the resistance to environmental factors such as heat and wetting, UV and SO_2_ [15], and freezing and thawing [16], which determine the façade’s life [17].

The results of many studies on weathering resistance have revealed that an ETICS developed systemically, that is, using the same thermal insulation material all over the facade, is characterised by high resistance to defects that occur as a result of exposure to environmental factors [18,19], while maintaining a constant level of performance [20,21]. An ETICS complying with EAD 040083-00-0404 is assumed to have maintained its performance for at least 25 years [7,22]. Considering the differences in the physical and mechanical characteristics of EPS and MW [11], including but not limited to their different thermal conductivities [18], linear expansions [23], absorbability, and diffusion resistance [6], which affect thermal insulation characteristics [24,25], the risk of damage in thermal insulation systems combining EPS and MW has been identified. Potential faults include discolouration [26,27] and cracks [26,28], which can lead to a loss in watertightness [29] and, consequently, in the building utility [30].

Previous studies on thermal insulation systems with fire barriers have focused on evaluating the impact of MW strips between storeys on fire spread over the façade [9,10]. The weathering resistance of non-systematic thermal insulation systems that combine EPS and MW has not been sufficiently recognised. Norvaisiene et al. [27] studied EPS-based thermal insulation systems with MW strips between the storeys and above the windows after weathering according to Guideline for European Technical Approval ETAG 004 [31], involving heating and wetting and heating and cooling. They observed no defects in the finishing layer composed of 1 mm-thick acrylic, silicate, and silicone finishing coats or a 2 mm-thick mineral finishing coat. However, they observed a 50% reduction in the adhesion of the rendering system to the thermal insulation material in the contact area of the thermal insulation materials. Krause et al. [32] made comparable observations in their study of a similar system after the same weathering exposition. They did not observe any scratches, cracks, blistering, or flaking in the finishing layer. It is important to emphasise that the weathering applied in both studies covered one sequence of hydrothermal cycles, including 80 heating and wetting cycles and five heating and cooling cycles. Freezing, thawing, and extended exposure were not considered in this study. This is a standard sequence for ETA-granting procedures.

**Figure 1:**
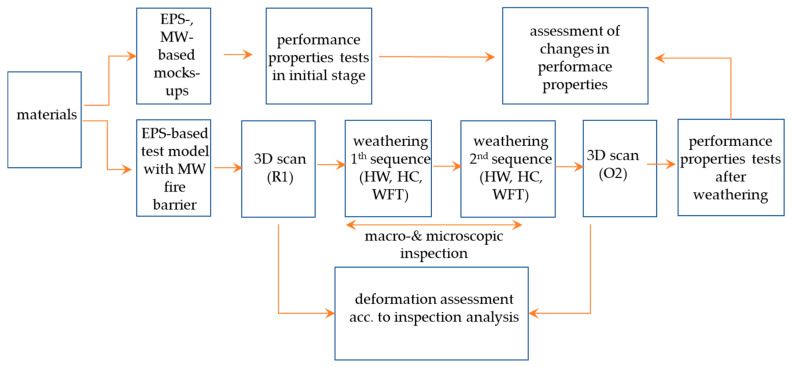
Experiment scheme.

An analysis of the climate data conducted by Barreira et al. [16], Kvande et al. [20], and Maia et al. [33] revealed that building facades in Europe, especially western façades, are exposed to much more diversified and intensive environmental impacts than previously considered. This is the result of climate change. Particular attention should be paid to precipitation severity and intensity, an increase in the mean annual temperature and the number of cases crossing the 0 °C threshold [34], and sensitivity for cement-based products [35,36] that are the primary component of rendering systems. In view of climate change, a higher intensity of exposure in accelerated tests seems justified, especially when non-standard solutions are tested, such as thermal insulation systems combining EPS and MW. Long-term observations previously did not cover external thermal insulation systems with fire barriers. Garecki et al. [26], in their studies on the impact of strengthening the reinforcement layer at the EPS-MW joint, typical for thermal insulation upper zones in high-rise buildings (>25 m), demonstrated that exposing the system twice to hydrothermal cycles, according to ETAG 004, caused cracks in the rendering system, both in the zone with single and double fibreglass mesh layers. In studies on a large-size testing model (2.5 m× 3.5 m), four types of renders, two EPS and MW localisation configurations and two mesh types were used. This resulted in the observation fields being reduced to 0.15–0.3 m^2^, which made it difficult to draw conclusions about the rendering system’s behaviour with continuous layers, typical for real conditions. This study indicates a problem with the resistance of EPS-MW joints in high-rise buildings in the 25th metre zone; however, the results cannot be directly related to the fire barrier area owing to significant differences in the way they are constructed. This study aimed to investigate the behaviour of an EPS-based thermal insulation system with a MW strip as a fire barrier, exposed to the long-lasting impact of environmental factors which had not been studied before. In view of climate change, nonconventional long-lasting exposure simulating the effects of intensive atmospheric factors was applied. Two exposure sequences, each covering 80 cycles of heating and wetting (HW), five cycles of heating and cooling (HC), and 30 cycles of wetting, freezing, and thawing (WFT), were performed. The set of exposures considering wetting, freezing, and thawing cycles has not been considered for ETICS with fire barriers. Changes in the finishing layer were monitored macroscopically and microscopically. A new approach to 3D scanning with inspection analysis was used to evaluate the deformation. The impact of the set exposures on the microstructure was analysed using an optical microscope and a scanning microscope. The effect on water absorption, water vapour permeability, and the bond strength between the rendering system and insulation product, as well as the tensile strength, are crucial for a thermal insulation system’s performance. Figure 1 shows the experimental scheme.

## 2. Materials and Methods

### 2.1. Materials

The thermal insulation system used for the tests was based on an ETICS configured using only an EPS. A purely bonded ETICS available in the market, covered by the current ETA, was used for the tests. It was modified by introducing a non-systemic mineral wool strip as a fire barrier, and the highest possible thickness (150 mm) of the thermal insulation material was used, taking into account the need to maintain an appropriate distance between the model and heat radiators in the chamber.

The EPS was fixed to the substrate using cement-based adhesive without auxiliary mechanical fixing. The same type of glue and universal facade dowels with an 8 mm diameter, 215 mm-long steel stem, and a 60 mm-diameter plastic plate were used to fix the mineral wool. The same type of finishing layer was developed for the entire thermal insulation system; the finishing layer consisted of a cement-based adhesive with an embedded single layer of fibreglass mesh, primer, and thin-coat render. Mineral thin-coat render is the most susceptible to environmental factors [19,37]. Data on the materials used in the thermal insulation system are summarised in Table 1.

### 2.2. Test Objects

The following were laboratory-prepared for testing.

- 3.6 m-ide and 2.8 m-high one-test model representing the thermal insulation system,
- 1.0 m × 0.5 m mock-ups representing fragments of the test model, that is, a thermal insulation system on an EPS layer (two mock-ups) and strips between the storeys on an MW layer (two mock-ups).

**Figure 2:**
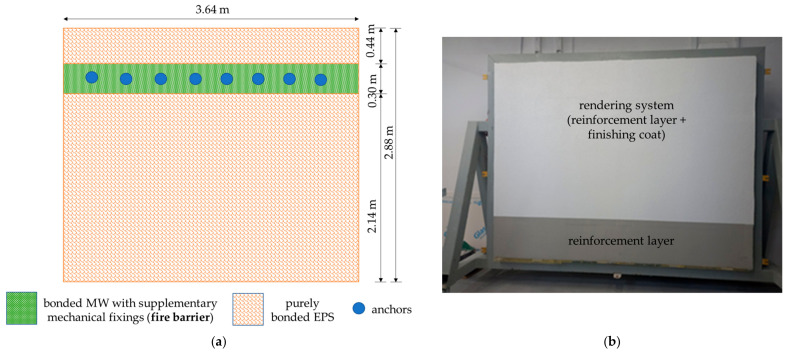
Test model: (**a**) schematic arrangement of thermal insulation materials, (**b**) view after applying the rendering, a base coat strip at the bottom.

Recommendations [8] for creating fire barriers were considered when designing the test model. Strips with a width of 300 mm were used (Figure 2a). The test model was developed according to ETICS recommendations [7,39]. Initially, thermal insulation (Table 1) was applied to the substrate, which was a masonry wall of aerated concrete blocks founded on a steel supporting structure that was indispensable for mounting the model in a weathering chamber. EPS boards were glued to the substrate with cement-based adhesive using the circumferential spot method. The same adhesive was used to fix the mineral wool; it was applied all over the surface of the boards and dowels were spaced every 450 mm (Figure 2a).

**Figure 3:**
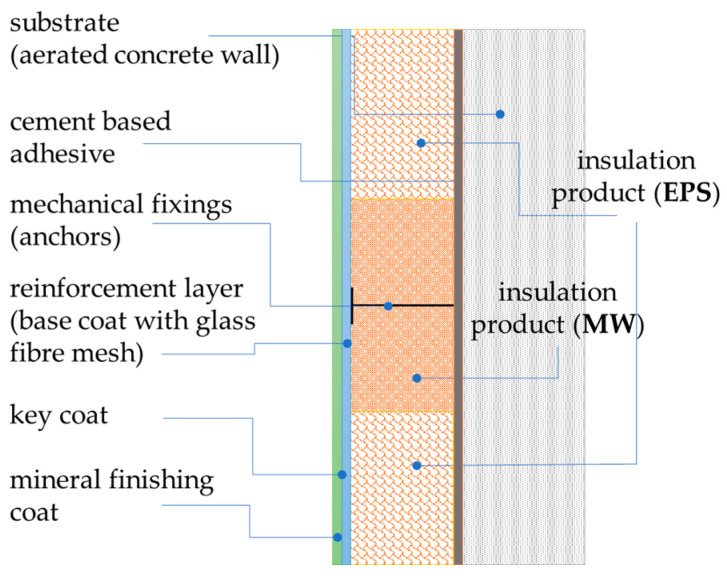
Test model—arrangement of layers.

A reinforcement layer (Table 1) of the cement-based adhesive with an embedded single layer of fibreglass mesh was applied continuously to a thermal insulation layer. No additional strengthening of the reinforcement layer was used, for example, another mesh layer. The reinforcement layer was approximately 5 mm thick. After drying, the soil was primed and weathered for a few days under laboratory conditions. Then, textured rendering was applied, skipping a 1.0 m-high control strip at the bottom (Figure 2b). The rendering layer was ca. 1.5 mm thick. The layers in the test model are schematically shown in Figure 3.

Mock-ups were prepared simultaneously to test the thermal insulation system under the original conditions. The layer arrangement in the mock-ups corresponds to that used in the test model, skipping the mechanical connectors. Two mock-ups were developed on the EPS layer, and the other two on the MW layer. Mock-ups were used to obtain the performance properties under the initial condition.

**Figure 4:**
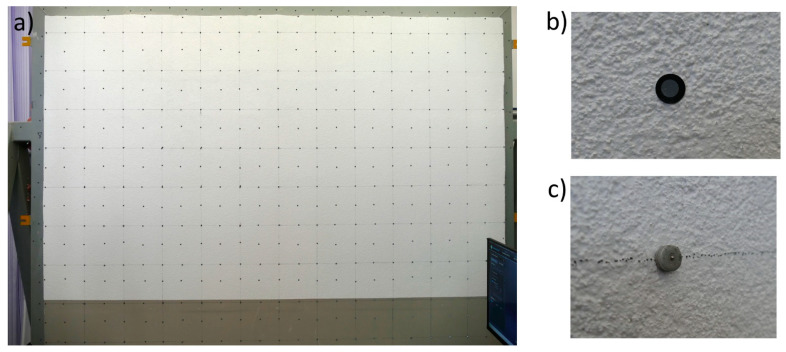
Mesh of identification points used for 3D scan positioning: (**a**) general view of the model, (**b**) marker, (**c**) benchmark.

The test model and mock-ups were seasoned for 28 days under laboratory conditions (T 23 ± 2 °C, RH 50 ± 5%). Then, a network of identification points (markers and benchmarks) was applied to the test model and used for the superficial positioning of the 3D scan (Figure 4a). The markers (Figure 4b) were applied to a 300 mm × 300 mm modular base mesh with replenishments at the edges and in the middle to create the target mesh. The vertical and horizontal distances between benchmarks (Figure 4c) were 600 mm.

### 2.3. Weathering

Weathering exposure was designed considering the observation results of environmental exposure to which building façades in Europe are subjected [16,20,33,34], as well as previous experiences in the weathering tests of ETICS [35,36], based on the standard ETA procedure sequence [7,31]. Standard ETICS testing hydrothermal cycles [31] were supplemented with a wetting, freezing, and thawing cycle series, typically used within a more limited scope and only for systems with absorbability > 0.5 kg/m^2^ [7]. Two exposure sequences were performed, with each covering

- 29-day-long hydrothermal cycles involving:
  – 80 cycles of heating and wetting (HW), 6 h each, including:
    · heating to T 70 ± 2 °C for 1 h,
    · maintaining T 70 ± 5 °C, RH ≤ 30% for 2 h,
    · wetting by sprinkling water at 15 ± 5 °C for 1 h,
    · drying for 2 h at 20 ± 5 °C,
  – conditioning for 48 h at T 20 ± 5 °C, RH ≥ 50%;
  – 5 cycles of heating and cooling (HC), 24 h each, were performed as follows:
    · heating to T 50 ± 2 °C for 1 h,
    · maintaining T 50 ± 5 °C and RH ≤ 30% for 7 h,
    · cooling down to T −20 ± 3 °C for 2 h,
    · freezing for 14 h at T −20 ± 3 °C,
  – conditioning for 48 h at T 20 ± 5 °C and RH ≥ 50%.
- 17-day-long wetting, freezing, and thawing (WFT), including:
  – water sprinkling at 15 ± 5 °C for 8 h;
  – 30 cycles, 8 h, including:
    · cooling down to T −20 ± 3 °C for 2 h,
    · freezing for 4 h at T −20 ± 3 °C,
    · thawing at T 20 ± 5 °C for 1 h,
    · water sprinkling at T 15 ± 5 °C for 1 h;
  – conditioning for 7 d at T 20 ± 5 °C and RH ≥ 50%.

**Figure 5:**
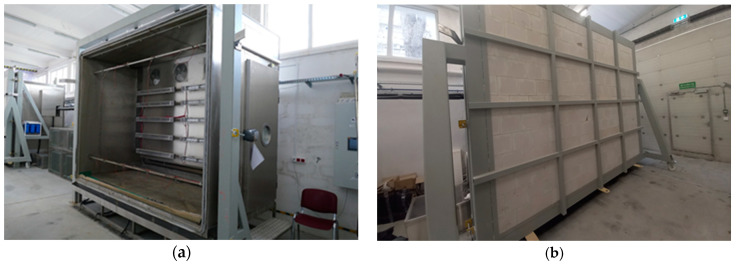
Artificial weathering chamber, view: (**a**) before assembling the test model (exposed heat radiators and spray nozzles), (**b**) after installing the test model exposed (aerated concrete wall as a substrate).

Weathering was performed in a climate chamber (UniMors, Warsaw, Poland) equipped with five heat radiators, two spray nozzles to ensure uniform water distribution (Figure 5a), and a cooling device. The test model was exposed to a positive temperature at a set level of relative humidity, wetting by water sprinkling, and a negative temperature. The test apparatus was positioned against the front face of the specimen (Figure 5b). The exposures were conducted automatically and continuously for 46 days in total for each sequence, and the conditions in the chamber were monitored every 1 h.

### 2.4. Macroscopic and Microscopic Inspection

The finishing layer was evaluated macroscopically and microscopically before and after each HW, HC, and WFT stage. It was checked whether defects visible to the naked eye were formed, including blistering or peeling of rendering systems (base coat and finishing coat), failure or cracking within the joints between thermal insulation boards, and detachment of the rendering systems or cracks [21].

Simultaneously, a microscopic assessment was conducted using a digital optical microscope (Delta Optical, Poland, Mińsk Mazowiecki) at a magnification of ×300, using Delta Optical Smart Analysis PRO software Genetic ProX1_2019 (Delta Optical, Poland, Mińsk Mazowiecki). A portable microscope helped inspect the finishing layer’s condition without the need to cut out samples and consequently did not compromise its structure. Cracks were primarily analysed for their course and width; differentiation was made between the crack sizes of ≤0.2 mm and >0.2 mm that enable water penetration to the thermal insulating layer [29].

After weathering, finishing layer samples were collected for microstructural analysis using a scanning electron microscope. The microstructure was investigated using a Sigma 500 VP scanning electron microscope with cold-field emission (Carl Zeiss Microscopy GmbH, Köln, Germany). The tests were carried out at an accelerating voltage of 10 keV with an excitation electron bundle using an SE detector on samples with a sprayed gold coat. The observations were performed at magnifications of ×500, ×5000, and ×10,000. The original samples collected from the test models were analysed in the same manner.

### 2.5. 3D Scanning with Inspection Analysis

**Figure 6:**
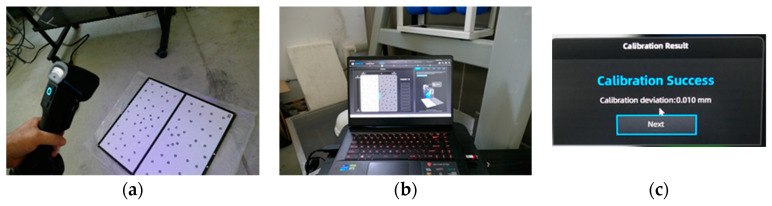
Scanner calibration: (**a**) calibration plate, (**b**) control software, (**c**) calibration result.

A 3D imaging was performed on the original model after weathering. A 3D FreeScan UEPro (SHINING 3D Tech Co., Ltd., Hangzhou, China) manual scanner with photogrammetry mode was used [40]. This is a metrological-grade scanner with the following parameters: light source—blue laser, 26 cross lines, scanning accuracy up to 0.02 mm, volumetric accuracy in the photogrammetry mode 0.02 mm + 0.015 mm/m, scanning rate 1,850,000 points/s. Before imaging, the environmental conditions were recorded, that is, temperature and air relative humidity; the device was calibrated using a dedicated plate as a template, and exposure to the scanner’s light was performed in various directions. The calibration results are shown in Figure 6.

Markers and benchmarks were applied to the wall surface and steel frame. The markers were used to position the scan on the surface and were used as a standard solution for imaging. Benchmarks were used for volumetric identification of the scans to help their compilation. This is a useful solution in inspection engineering. The ETICS thermal insulation finishing layer with a steel-mounting frame was then scanned. The frame was used as a set of reference surfaces to position the scans in the inspection software. The model in its original state (R1 reference scan) was scanned at T 20.2 °C and RH 51.6%. Post-weathering scanning (O2 operating scan) was performed at T 22.5 °C and RH 69.9%.

The data obtained through scanning were subjected to inspection to identify qualitative and quantitative differences in the geometry of the reference and operating models. A 3D computer-aided design (CAD) model or model template scan, which is a reference for all measurements of real models, can be used as the reference model. In this case, the scan of the test object was the reference model. Typically, an operating model is a scan of an item with a potentially disturbed geometry (e.g., in production) that differentiates it from the reference model. The reference and operating models were combined into pairs, where the first was the benchmark for matching the operating object. Matching was performed according to set rules, indicating shared areas. In the analysed case, the shared areas included a steel frame with a mounted thermal insulation system model. The steel frame was assumed to be a robust item with side stiffening components (angle struts) and disc stiffening in the wall plane, where the thermal insulation system was fixed. The test model with the frame was placed in an air-conditioned laboratory with controlled temperature and humidity. Consequently, the changes in frame geometry due to exposure to these factors can be considered negligible. The inspection data were obtained from the R1 reference model and O2 operating model scans. This modification involves distorting the model geometry as a result of weathering. Matching the reference and operating scans helped obtain inspection data (INS). The inspection analysis was performed usingGeomagic Control X software (Build Version: 2024.1.0; Build Number: 65).

### 2.6. Performance Tests

The performance of the original samples, samples from mock-ups, and those obtained from the test model was evaluated.

The water absorption of the rendering system (base coat and finishing coat) was tested according to EAD 040083-00-0404 [7] on 200 mm × 200 mm samples covering the rendering system and the thermal insulation product. The edges of the samples were protected with epoxy resin and then subjected to three cycles, each including immersion in water for 24 h and drying in a forced-air circulation dryer for 24 h at T 50 ± 5 °C. After the cycles, the samples were stored for 24 h under laboratory conditions (23 ± 2 °C and 50 ± 5% RH) and then immersed in water to a depth of 2 mm below the water surface with the rendering system downwards. The samples were weighed 3 min, 1 h, and 24 h after immersion. Water absorption (*WA*) was calculated as the ratio of the weight of the sample before and after immersion in water to the area of the sample; the result is expressed in kg/m^2^. Three samples were tested for each series.

Water vapour permeability was tested according to EAD 040083-00-0404 [7] using the wet cup method. After separating the thermal insulation material, discs with ca. 5000 mm^2^ areas were cut out from the rendering system. Original samples were conditioned according to the ISO 7783 [41], B method, consisting of three cycles, each involving immersion in water at 23 ± 2 °C for 24 h and drying at 50 ± 2 °C for 24 h. The discs were fixed in cups with an ammonium dihydrogen phosphate (NH_4_H_2_PO_4_) solution in distilled water. The cups were sealed in liquid paraffin. The tests were conducted under laboratory conditions. The diffusion equivalent of the air layer thickness s_d_, was calculated according to (1) and expressed as m. Five samples were tested for each series.

(1) $$s_{d}=\frac{\delta_{a}\times {\Delta p}_{v}}{V}$$

where *δ_a_* is the coefficient of air water vapour permeability (at T 23 °C and standard pressure (101,325 Pa); the coefficient value is 0.0169 g/(m⋅d⋅Pa)), Δ*p_v_* is the difference (Pa) between the water vapour partial pressure in the test vessel and the pressure in the test chamber (i.e., between two sides of the tested sample), and *V* is the water vapour permeability rate in g/m^2^ a day.

The resistance to hard body impact test was performed according to ISO 7892 [42]. Steel balls weighing 0.5 kg and 1.0 kg were used. The balls were dropped from a height ranging from 0.61 m to 1.02 m, appropriate to the adopted impact energy of 3 J or 10 J. Before the test, the original samples were immersed in water for seven days and then seasoned for seven days under laboratory conditions according to the guidelines [7]. The test was performed after seven days of seasoning in the test model under laboratory conditions. The test began with 3 J impact energy (0.5 kg ball, 0.61 m fall height), and if the sample was not damaged, the test continued while increasing the impact energy until the damage occurred. Any cracks, scaling, or debonding between the layers visible to the naked eye were considered damage. The damage size was measured using callipers. Any possible permanent deformation not accompanied by the above-mentioned features was not considered as damage. The test result was the maximum impact energy at which no damage was found in the five tests.

The bond strength between the rendering system and the thermal insulation material was tested using a pull-off method according to EAD 040083-00-0404 [7]. Steel holders (50 mm × 50 mm) were glued to the finishing layer using epoxy resin. Then, an incision was made to the full height of the finishing layer up to the thermal insulation layer along the holder edges. After 48 h of seasoning, stretching was performed with a force perpendicular to the thermal insulation plane. The tests were performed using a class 1 computer-controlled tensile testing machine (Instron, Darmstadt, Germany), with a constant head travel rate of 10 ± 1 mm/min. The force values were recorded until damage occurred. The bond strength *σ_B_* was calculated as the quotient of the maximum recorded bond force and cross-sectional area of the test sample, expressed in kPa. Six samples were tested in each series.

The tensile test of the rendering system was carried out on samples obtained from the original mineral-wool-based panels and on the test model after weathering. The test model samples were collected vertically from the MW fire barrier area, EPS area near the fire barrier, and MW and EPS contact areas. The finishing layer was separated from the thermal insulation material, and then 600 mm-long and 50 mm-wide strips were cut out from the finishing layer. The thicknesses of the samples ranged from 5.1 to 5.7 mm. After 48 h of seasoning under laboratory conditions, a tensile test was conducted with the force acting within the plane of the finishing layer. A class 1 computer-controlled tensile testing machine (Zwick Roell, Germany, Ulm) was used with a constant head travel rate of 2 mm/min. The force value until the damage and displacement values at the 100 mm section were recorded. The tensile strength *σ_T_* was calculated as the maximum recorded tensile force and cross-sectional area of the test sample, expressed in MPa, and the elongation at break was expressed as a percentage. Six samples were tested in each series.

## 3. Results and Discussion

### 3.1. Macroscopic and Microscopic Assessment

**Figure 7:**
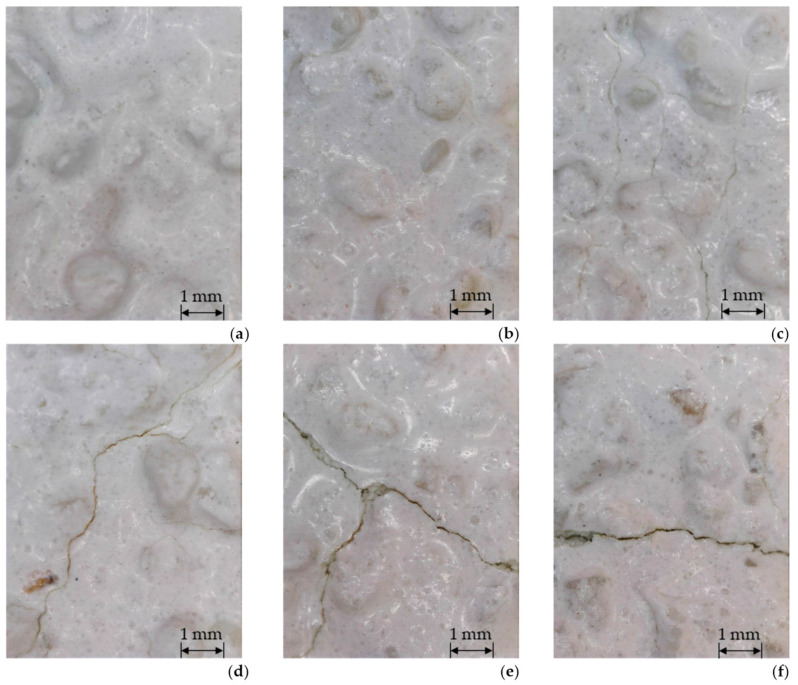
View of the rendering system: (**a**) in its original condition; (**b**) after heating and wetting and heating and cooling in the first exposure sequence; (**c**) cracks ≤ 0.2 mm, primarily within the fire barrier, after wetting, freezing, and thawing in the first exposure sequence; (**d**), cracks ≤ 0.2 mm observed all over the test model’s surface after wetting, freezing, and thawing in the second exposure sequence; (**e**,**f**) cracks > 0.2 mm within the fire barrier after wetting, freezing, and thawing in the second exposure sequence; magnification ×300.

Macroscopic assessment of the original state of the thermal insulation system model revealed that the finishing layer was white, textured, and rough characteristically for a 1.5 mm grain size distribution in the render. No failures or damages were observed in the macroscopic or microscopic assessments (Figure 7a). This assessment was repeated after each weathering stage. The results of the macroscopic assessment are summarised in Table 2. Neither blistering, peeling, nor detachment of the rendering systems was observed at any exposure stage, which corresponds to the results of Norvaisiene et al. [27] and Krause et al. [32].

Cracks dominated among the defects and were not observed (Figure 7b) after the first heating and wetting (HW) and heating and cooling (HC) series, that is, standard exposures in ETICS performance assessment for the ETA needs [7]. Norvaisiene et al. [27] and Krause et al. [32] ended their tests at this stage, not having observed any defects. The first cracks became visible after the last stage of the first exposure sequence, involving wetting, freezing, and thawing (WFT). Only a few cracks were observed, mainly within the fire barrier-simulating strip, and the cracks were primarily non-directional. Their width did not exceed 0.2 mm (Figure 7c). This exposure stage was among the first stages to exceed the standard testing range. It is used, to a limited extent (five cycles), for ETICS with the rendering’s water absorption rate of >0.5 kg/m^2^ [7], but in the future, it may become obligatory for all systems. A higher intensity of cracks was observed during the successive exposure stages. After the last stage of the second exposure sequence, cracks ≤ 0.2 mm were observed on almost the entire surface of the test model in moderate numbers and mainly non-directional (Figure 7d). The above matches the observations of thin-coat rendering behaviour as a result of long-term exposure presented in other studies. Microcracks have been observed over extended weathering exposure periods, where they have typically been limited to the binder-aggregate phase border [35,37,42]. A higher density of nondirectional cracks was observed in the strip simulating the fire barrier and near the contact area of the two thermal insulation products, that is, MW and EPS, where the cracks formed a dense pattern. Numerous cracks > 0.2 mm (Figure 7e) were identified in these areas. Some were non-directional, whereas others were almost horizontal (Figure 7f). Crack widths > 0.2 mm suggest a loss of the barrier function of the finishing layer for water [19,28], which is a determinant of ETICS life [20,22].

**Figure 8:**
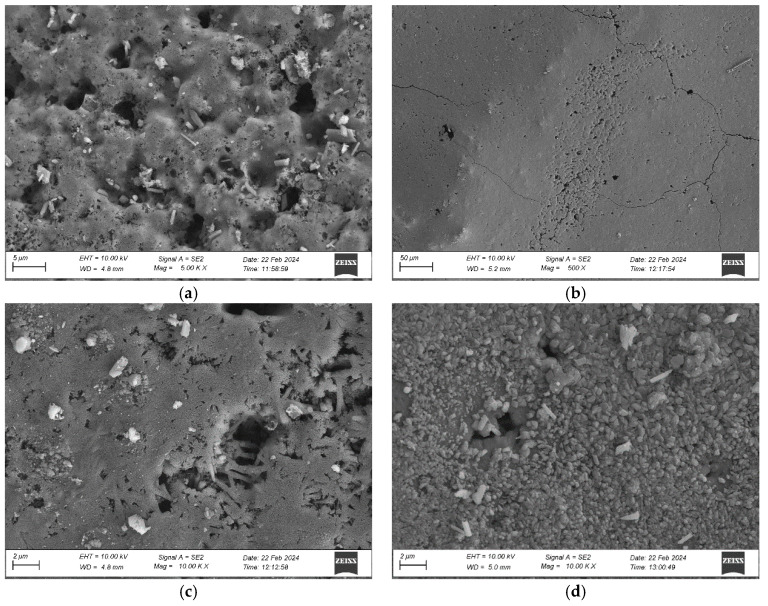
Rendering system’s surface morphology: (**a**) numerous macropores on the original render’s surface (×5000), (**b**) cracks after weathering (×500), (**c**) macropores on the original render’s surface (×10,000), (**d**) calcite CaCO_3_ on the render’s surface after weathering (×10,000).

The rendering system was analysed microscopically using SEM. Observations of the original sample revealed a well-developed surface with numerous open pores (Figure 8a). Open macropores with diameters ranging from 1 µm to 5 µm prevailed. No mineral precipitation was detected; only contaminant particles most likely originated from the thermal insulation material. The particles could have been applied to render while preparing the thermal insulation system samples. The analysis of the render after weathering, carried out at a small magnification (Figure 8b), revealed cracks which had a course and width similar to those observed with an optical microscope (Figure 7e,f). A magnification of ×10,000 revealed the differences in the surface morphology. In the original condition, only open pores and contaminants were visible (Figure 8c), whereas after weathering, some pores and capillaries were covered with crystalline material (Figure 8d), most likely calcium carbonate CaCO_3_ [35]. Calcite often appears on the mineral rendering surface owing to exposure to environmental factors [43,44]. Exposure to water triggers calcium hydration [43]. The crystals can contribute to reducing the pore sizes, as observed in the analysed case (Figure 8c,d). This phenomenon negatively affects the adhesion forces in the render-base coat contact zone and may lead to delamination of the thin-coat render [35,45].

### 3.2. Inspection Analysis

**Figure 9:**
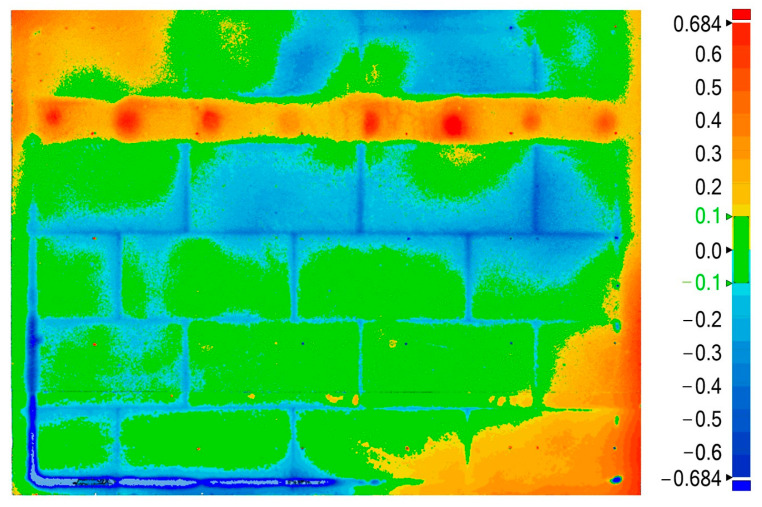
Map of the thermal insulation system deformations after weathering versus the original state (based on an inspection analysis of the R1-O2 pair; deformation expressed in mm).

The data obtained by scanning the original and weathered models, covering two sequences of heating and wetting (HW); heating and cooling (HC); and wetting, freezing, and thawing (WFT), were subjected to inspection analysis involving the identification of quantitative and qualitative differences in the geometries of the reference and operating models [40]. The results of the inspection analysis are shown in Figure 9 as a map of the deformations of the finishing layer of the thermal insulation system. Deformation is described as the displacement difference between the wall after weathering (operating model) and that before exposure (reference model). The deformation direction was read perpendicular to the surface of the wall. Green indicates that the result is within the acceptable tolerance range (±0.1 mm), corresponding to the accuracy of the device for the analysis.

Deformation *D* is determined as a scalar value according to Equation (2).

(2) $$D=\sqrt{{GV}_{x}^{2}+{GV}_{x}^{2}+{GV}_{z}^{2\;}}$$

The *GV* is determined as the difference between the position of the point on the stereolithography (STL) mesh of the operating model (after deformation) versus the reference model (before deformation). The vectors were determined using the adopted reference system. In this case, it was normally perpendicular to the reference surface. Generally, reference points are defined as follows:

(3) $$P_{o}=\langle x_{0}\;{,y}_{0}\;{,z}_{0}\;\rangle ;\;P_{r}=\langle x_{r}\;{,y}_{r}\;{,z}_{r}\;\rangle$$

where *x*, *y*, and *z* are the node’s coordinates; *o*—operating model, *r*—reference model.

Vector:

(4) $$GV=\langle x_{o}-x_{r},\;y_{o}-y_{r}{,\;z}_{o}-z_{r}\rangle$$

The fields marked in Figure 9 in yellow to red hues represent positive deformation, which is the uplift of the wall’s surface. Blue hues represent negative deformation, which is surface concavity. Green represents the tolerance area (±0.1 mm) to which the neutral results belong, and the surface is flat.

Analysis of the above data revealed an apparent positive deformation within the fire barrier. All over the surface of the MW strip 0.3–0.5 mm uplifts were observed, with extreme values up to 0.7 mm where mechanical joints (red) were found. The dowel plastic plates (Section 2.1) seemed to have been deformed under the set exposure. The deformation was transferred to the finishing layer, which was not observed in other studies [46]. Outside the MW strip area, a rapid transition into a concavity zone is marked in blue, where a negative deformation of 0.2–0.6 mm was recorded. The largest deformation difference occurred directly in the two zones, with a total value of up to 1.2 mm. In the area of the fire barrier and the zone around the MW strip with the highest deformation difference, the highest intensity of cracks was observed. Crack sizes of >0.2 mm (Figure 7e,f) suggest a loss of tightness of the rendering system [19,28].

Some deformation disturbances towards bulging were observed at the corners. Noticeable contact points of the EPS boards (horizontal and vertical blue lines) are another original effect observed in the concavity zone.

### 3.3. Performance

The original samples and those after full weathering were tested for the thermal insulation system’s performance in terms of water absorption, water vapour permeability, resistance to hard body impact, bond strength between the rendering system and thermal insulation material, and tensile strength, covering two sequences: heating and wetting (HW), heating and cooling (HC), and wetting, freezing, and thawing (WFT).

**Figure 10:**
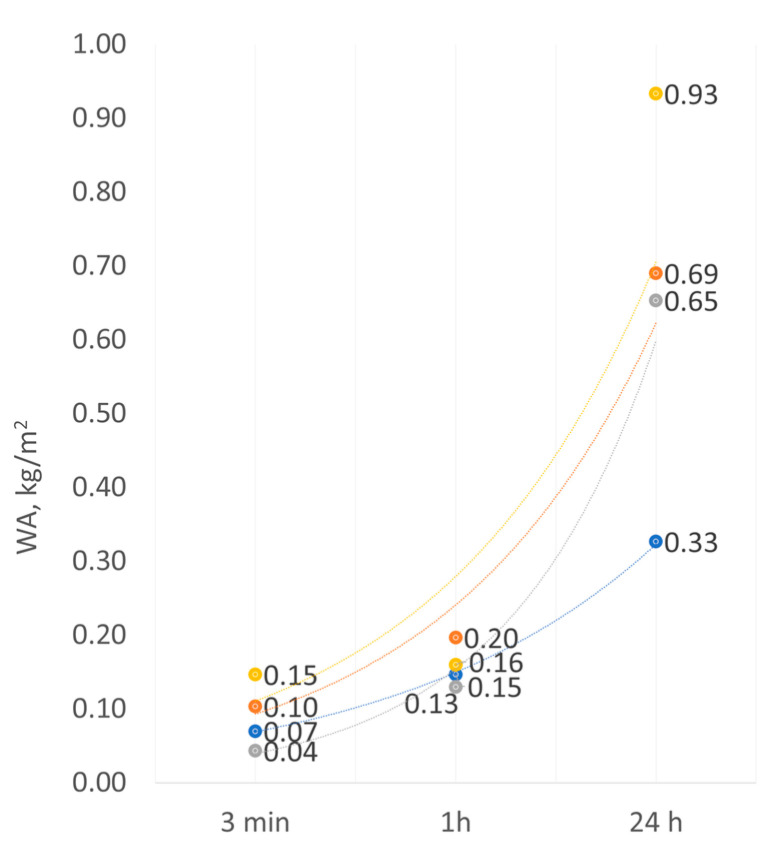
Water absorption of the rendering system after 3 min, 1 h, and 24 h of immersion in water; EPS-based system: 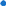 original condition, 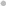 after weathering; MW-based system: 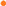 original condition, 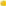 after weathering.

Water absorption by an EPS-based rendering system amounted to 0.33 kg/m^2^ in the original condition and 0.65 kg/m^2^ after weathering after 24 h of immersion in water (Figure 10). The MW-based rendering system reached the values of 0.69 kg/m^2^ and 0.93 kg/m^2^, respectively. Thus, it can be concluded that weathering increased the absorption of the EPS-based systems by nearly 50% and 25%, respectively. The increase in absorption can be caused by a change in the porosity of the render. According to Bochen [35] and Ślusarek et al. [36], owing to exposure to environmental factors, the open porosity of the mineral render increases to ca. 17% despite carbonation, which leads to calcite (CaCO_3_) formation. This was observed in the surface morphology analysis (Figure 8d). In addition to the increase in open porosity, numerous cracks in the rendering system, including those wider than 0.2 mm (Figure 7e,f), cause a loss in the watertightness of the finishing layer [19,28]. Thus, it can be concluded that after weathering, water was transported into further thermal insulation layers not only through the channels created by the pores [25], but also by the cracks. After weathering, the absorption of the MW-based system was more than 50% higher than that of the EPS-based system. These differences can be attributed to the differences in the susceptibility of the thermal insulation materials to water impact [11]. Similar relationships were observed by Norvaišienė et al. [27]. Moreover, one should pay attention to the fact that a thermal insulation system with absorption over 0.5 kg/m^2^ should be considered sensitive to freezing-thawing [7].

**Figure 11:**
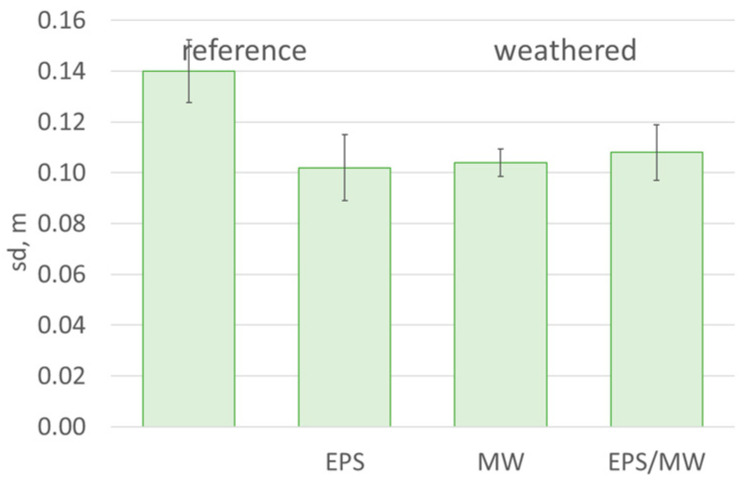
Diffusion-equivalent air layer thickness s_d_ of the rendering system in the original condition and after weathering; the error bars represent standard deviation.

According to the literature, the macropore radii in mineral rendering are over 1000 nm [43], which explains their high vapour permeability, which is even higher than that of acrylic or silicone rendering [35]. The tested rendering system with mineral render was characterised by a very low diffusion resistance. The diffusion-equivalent air layer thickness s_d_ determined in the tests amounted only to 0.14 m (Figure 11) in the original condition. To ensure ETICS functionality, values of up to 2 m are accepted for EPS-based systems and up to 1 m for MW-based systems [7]. The results of previous studies have also revealed that pores in minerals render regroups during weathering. Consequently, nearly 80% are sub macropores with diameters ranging from 100 to 1000 nm, whereas 15% are mesopores with diameters up to 100 nm [35,36]. The size of the pores significantly affects the material properties, primarily their interaction with water. The results of previous studies suggest that mesopores are responsible for the volume and rate of water vapour transport [25]. This is reflected in the changing trend of the diffusion equivalent air layer thickness s_d_, which decreases slightly after weathering. As a result of the set weathering, the finishing layer revealed that water permeability tends to increase, which should be considered beneficial from the point of view of a thermal insulation system’s functionality [23,25].

**Figure 12:**
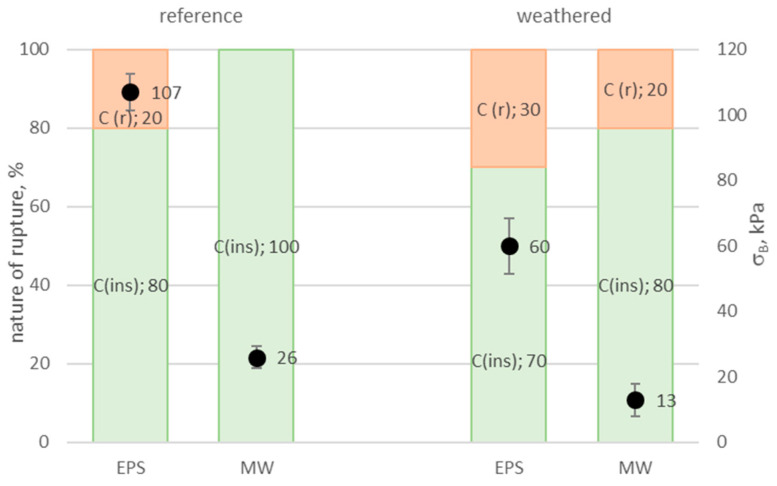
Rendering system’s adhesion to EPS and MW, expressed as • bond strength σ_B_ and nature of damage: C—cohesive damage within 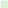 insulation product (ins), 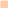 render (r); the error bars represent the standard deviation of the bond strength.

The bond strength between the thermal insulation system layer, including the rendering system and the insulation product, is among the critical performance characteristics of ETICS [4]. It is assumed that adhesion should be no less than 80 kPa, whereby cohesive or adhesive rupture or damage should occur within the thermal insulation product in a fully cohesive way (100%) [7]. In the tested solution, the bond strength between the rendering system and the EPS was 107 kPa in the original state at 80% cohesive damage within the insulation product. For MW, the bond strength was 26 kPa at full (100%) cohesive damage within the insulation product (Figure 12). The obtained bond strength values corresponded to the perpendicular tensile strength of the applied insulation products (Table 1). Norvaišienė et al. [27], Krause et al. [32], and Malanho et al. [45] made similar observations in their studies conducted using standard hydrothermal cycles, covering one sequence of heating and wetting (HW), heating and cooling (HC), and five cycles of wetting, freezing, and thawing (WFT).

In this study, after long-lasting weathering, the bond between the EPS- and MW-based rendering systems decreased by 60 and 50%, respectively. Such a deterioration in the bond strength between the rendering system and the EPS and MW was not observed after standard cycles45]. Norvaišienė et al. [27] paid attention to the deteriorated bond strength in the EPS and MW contact area despite no damage to the finishing layer observed in the macroscopic assessment. In this study, a lower share of damage within the insulation products was also observed in favour of the render’s cohesive damage, which is non-characteristic of thermal insulation systems. The cohesive damage share amounted to 30% and 20% for a system with EPS and MW, respectively, which can suggest deteriorated strength of the rendering as a result of the set exposures [35]. However, no adhesion was observed between the thermal insulation layers. Considering the nature of the damage, mainly within the insulation product, and the observed level of breaking stress, it can be concluded that moisture penetrated the insulation products through the cracks in the rendering system (Figure 7), leading to deteriorated strength parameters of both the EPS and MW [11,18,45]. As a result of set weathering, the bond strength dropped significantly, and the bond strength determined the construction utility of a thermal insulation system [3].

**Figure 13:**
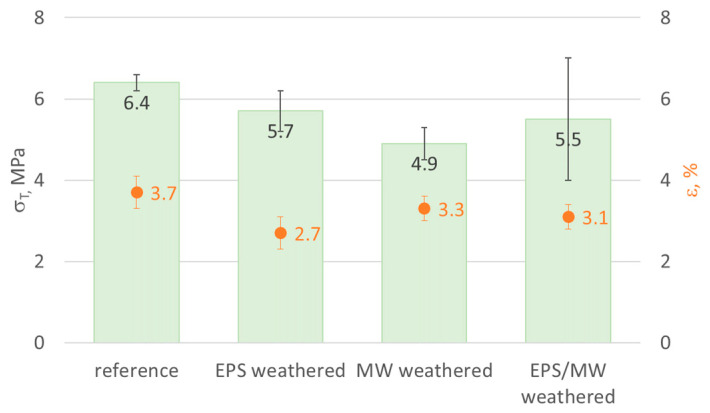
Rendering system behaviour at stretching: 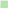 tensile strength σ_T_, 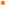 elongation at break ε; identified for the EPS- and MW-based rendering system and in the EPS/MW contact area; error bars represent standard deviation.

An analysis of the rendering system’s behaviour in the tensile test within the finishing layer plane revealed a 10% decrease in the strength after weathering for EPS-based areas and over 20% decrease for MW-based areas, with a simultaneous slight elasticity reduction demonstrated by lower values of elongation at break (Figure 13). The differences in the behaviour of the EPS- and MW-based rendering systems in the tensile test can result from the higher intensity of cracks in the fire barrier strip, which could weaken the stretched cross-section. Samples collected from the EPS and MW border areas were characterised by tensile strength values between those obtained from EPS- and MW-based areas, whereby the result range was much broader, i.e., from 3.1 to 7.2 MPa. This can result from the >0.2 mm wide cracks observed locally in the EPS and MW contact areas (Figure 7e,f). No delamination of the rendering system layers was observed during the tensile test.

Subjecting the thermal insulation system to hard-body impact, with 3 J impact energy, caused circular cracks with diameters of up to 33 mm in the EPS-based rendering system, with no penetration in the thermal insulation product. No deterioration was observed in the MW-based system. Increasing the impact energy to 10 J resulted in the formation of circular cracks with similar diameters and no MW penetration (Table 3). The above helped us classify an EPS-based system as impact resistance category III and an MW-based system as higher category II. After weathering, the impact resistance of the EPS-based system did not change, corresponding to [32]. The damage type and size at 3 J impact energy increased for the MW-based system, resulting in a classification reduction to category III. The EPS and MW contact areas behaved similarly, as observed by Norvaišien et al. [27]. In the absence of delamination between the finishing layer and the base coat and between the base coat and the insulation product, delamination within the base coat or exposed reinforcement was not observed.

Considering impact resistance class III, the tested solution can be regarded as suitable for use only in zones where risk occurs of damage by normal impacts caused by people or by thrown or kicked objects [7]. Nevertheless, despite considerably intensive weathering, the results remained at the characteristic level of ETICS with mineral rendering [25]. Thermal insulation systems with silicone or silicone-silicate rendering are characterised by higher impact resistance values, even up to 20 J [47]. Based on these results, it can be concluded that set weathering did not significantly affect the impact resistance of the tested thermal insulation system.

There are plans to continue research on the resistance of thermal insulation systems with fire barriers to intense exposure to environmental factors. They should consider the impact of rendering system reinforcement using an extra reinforcement mesh layer and substituting a cement-based base coat with dispersion products. This research will be extended to cover polymer-rendering systems. Moreover, the diagnosis of ETICS changes owing to exposure should be developed using 3D scanning with inspection analysis.

## 4. Conclusions

A thermal insulation system with a thin-coat mineral render, made with expanded polystyrene thermal insulation, was tested, and a non-systemic solution in the form of a mineral wool strip was introduced into the tested system.

An analysis of the results revealed that hydrothermal exposures executed with a standard method for testing low-absorbability ETICS (<0.5 kg/m^2^) did not damage the finishing layer. A macro- and microscopic assessment was carried out within the entire large-sized test model, paying particular attention to the area of the fire barrier and contact area of the two thermal insulation materials.

Completing weathering with freeze-thaw cycles, simulating crossing the 0 °C threshold typical of many regions in Europe, resulted in the formation of a low number of non-directional cracks in the rendering system. They were observed only in the fire barrier zone. The cracks were no wider than 0.2 mm, which was taken as the tightness criterion of the ETICS finishing layer.

Long-lasting weathering was performed, simulating exposure to severe atmospheric factors due to rapid and intensive climate change. Two sequences of heating-wetting, heating-cooling, and freeze-thawing resulted in intensified cracks, with their extended areas of occurrence covering the entire test model. Within the MW strip and the EPS–MW contact area, their pattern was dense, but their presence in the remaining part of the model was scarce. Within the barrier and in its vicinity, cracks wider than 0.2 mm were observed, suggesting that the rendering system lost its watertightness. An inspection analysis of the data collected under 3D scanning of the test model revealed deformation of the thermal insulation system owing to long-lasting weathering. This was most apparent within the MW strip, where the recorded positive deformation amounted to 0.7 mm.

Long-lasting weathering results in a significant deterioration in performance. Nevertheless, the recorded increase in water absorption was still acceptable for thermal insulation systems. Consequently, the bond strength reached a very low level. This was accompanied by a partial shift in the failure zone into the rendering system. Moreover, a decrease in the tensile strength of the rendering system was observed but with no layer delamination. No deterioration in water vapour permeability or impact resistance was observed.

The impact of the thermal insulation material type on the response of the rendering system to long-lasting weathering is evident. In the MW-based zone, the water absorption values were much higher, whereas the bond strength and tensile strength values were lower, which could be attributed to the intensity and size of the cracks in the referenced area.

In summary, it can be concluded that the tested EPS-based thermal insulation system with a mineral wool strip exhibited acceptable resistance to standard European-averaged hydrothermal exposures. Long-lasting weathering, attempting to simulate intensified environmental impacts, resulted in the rendering system losing its watertightness. This was followed by the deterioration of some critical performance characteristics. The set exposures clearly affected the MW strip and the MW–EPS contact zone.

## Figures and Tables

**Table 1:** Materials used in the thermal insulation system.

Thermal Insulation System Layer	Material Characteristics
Adhesive for fixing thermal insulation to the substrate	Dry mix of cement-based binder, aggregates and modifiers; grain size distribution <0.8 mm, bulk density 1520÷1860 kg/m^3^, less than 98.4% ash content at 450 °C, mixed with water
Insulation product:	
base (systemic solution)	Expanded polystyrene (EPS), according to EN 13163 [38], 150 mm thick, λ_D_ = 0.038 W/mK, EPS-EN 13163-T1-L2-W2-S2-P5-BS115-CS(10)70-TR100-DS(N)2-DS(70,-)2-R100 code, perpendicular tensile strength ≥ 110 kPa and tensile strength ≥ 39 kPa determined in tests
fire barrier	Mineral rock wool (MW) according to EN 13162 [12], 150 mm thick, λ_D_ = 0.035 W/mK, MW-EN 13162-T5-DS(70,90)-CS(10)20-TR20-PL(5)200-WS-WL(P)-MU1 code, perpendicular tensile strength ≥ 22 kPa and tensile strength ≥ 9 kPa determined in tests
Reinforcement layer:	
base coat	The same product as that used for fixing thermal insulation to the substrate
reinforcement	1000 mm-wide fibreglass mesh, mesh size 4.5 mm × 5.0 mm; surface mass 150 g/m^2^, characterised by residual resistance after alkali ageing ≥ 20 N/mm and relative residual resistance after alkali ageing ≥ 50%
Key coat	Universal primer for thin-coat mineral rendering (liquid), volumetric density 1368 ÷ 1672 kg/m^3^ and dry matter content 55.6 ÷ 64.4%
Finishing coat	Dry mix based on the microfibre-reinforced composition of mineral binder (high-alkaline potassium water glass), inorganic binders, coarse marble aggregates, silicate flour, modifiers and preserving agents, 1.5 grain size distribution bulk density 1320 ÷ 1800 kg/m^3^, less than 99.8% ash content at 450 °C, mixed with water.

**Table 2:** Results of macroscopic assessment of the finishing layer.

Defects	Exposure Stage ^(1)^					
	Sequence I			Sequence II		
	HW	HC	WFT	HW	HC	WFT
blistering or peeling	not found	not found	not found	not found	not found	not found
failure or cracking associated with joints between thermal insulation boards	not found	not found	not found	not found	not found	found
detachment of the rendering system	not found	not found	not found	not found	not found	not found
≤0.2 mm-wide cracks	not found	not found	found ^(2)^	found ^(3)^	found ^(3)^	found ^(3)^
>0.2 mm-wide cracks	not found	not found	not found	found ^(4)^	found ^(4)^	found ^(4)^

^(1)^ assessment after completing the stage; ^(2)^ primarily within the fire barrier, low number, non-directional; ^(3)^ all over the model’s surface; outside low number outside the fire barrier’s area and dense pattern near the contact areas of various thermal insulation products (MW and EPS); typically, non-directional; ^(4)^ within the fire barrier and near the contact areas of various thermal insulation products (MW and EPS); numerous, non-directional or horizontal.

**Table 3:** The finishing layer’s impact resistance.

Insulation Product	Impact Energy	Defects/Diameter of Damage, mm/ Wide of Cracks, mm	Impact Resistance Category
Reference samples			
EPS	3 J ^(1)^	not penetrated ^(3)^/25 ÷ 33/<0.2	III
MW	3 J	no deterioration ^(4)^	II
	10 J ^(2)^	not penetrated/28 ÷ 36/<0.2	
Weathered samples			
EPS	3 J	not penetrated/25 ÷ 33/<0.2	III
MW	3 J	not penetrated/24 ÷ 34/<0.2	III
EPS/MW	3 J	not penetrated/25 ÷ 34/<0.2	III

^(1)^ 0.5 kg ball, 0.61 m fall height; ^(2)^ 1.5 kg ball, 1.02 m fall height; ^(3)^ superficial damage, provided there was no cracking; ^(4)^ circular cracking not penetrating as far as the thermal insulation product was observed.

## Data Availability

Data are contained within the article.

## References

[1] Michałowski B., Marcinek M., Tomaszewska J., Czernik S., Piasecki M., Geryło R., Michalak J. (2020). Influence of Rendering Type on the Environmental Characteristics of Expanded Polystyrene-Based External Thermal Insulation Composite System. Buildings.

[2] Pasker R. (2017). The European ETICS market–Do ETICS sufficiently contribute to meet political objectives?. Proceedings of the 4th European ETICS Forum.

[3] Michalak J. (2021). External thermal insulation composite systems (ETICS) from industry and academia perspective. Sustainability.

[4] Sudoł E., Kozikowska E. (2021). Mechanical Properties of Polyurethane Adhesive Bonds in a Mineral Wool-Based External Thermal Insulation Composite System for Timber Frame Buildings. Materials.

[5] Michalak J., Czernik S., Marcinek M., Michałowski B. (2020). Environmental burdens of External Thermal Insulation Systems. Expanded Polystyrene vs. Mineral Wool: Case Study from Poland. Sustainability.

[6] Hung Anh L.D., Pásztory Z. (2021). An overview of factors influencing thermal conductivity of building insulation materials. J. Build. Eng..

[7] (2020). European Assessment Document External Thermal Insulation Composite Systems (ETICS) with Renderings.

[8] (2018). Design Guidelines: Insulation of Building Facades due to Fire Safety.

[9] Niziurska M., Wieczorek M., Borkowicz K. (2022). Fire Safety of External Thermal Insulation Systems (ETICS) in the Aspect of Sustainable Use of Natural Resources. Sustainability.

[10] Čolić A., Pečur I.B. (2020). Influence of Horizontal and Vertical Barriers on Fire Development for Ventilated Façades. Fire Technol..

[11] Schiavoni S., D’Alessandro F., Bianchi F., Asdrubali F. (2016). Insulation materials for the building sector: A review and comparative analysis. Renew. Sustain. Energy Rev..

[12] (2015). Thermal Insulation Products for Buildings. Factory Made Mineral Wool (MW) Products. Specification.

[13] (2019). Fire Classification of Construction Products and Building Elements. Part 1: Classification Using Data from Reaction to Fire Tests.

[14] Regulation (EU) No 305/2011 of the European Parliament and of the Council. https://eur-lex.europa.eu/legal-content/EN/TXT/?uri=uriserv:OJ.L_.2011.088.01.0005.01.ENG&toc=OJ:L:2011:088:TOC.

[15] Parracha J.L., Borsoi G., Veiga R., Flores-Colen I., Nunes L., Garcia A.R., Ilharco L.M., Dionísio A., Faria P. (2021). Effects of hygrothermal, UV and SO_2_ accelerated ageing on the durability of ETICS in urban environments. Build. Environ..

[16] Barreira E., de Freitas V.P. (2013). Experimental study of the hygrothermal behaviour of External Thermal Insulation Composite Systems (ETICS). Build. Environ..

[17] Tavares J., Silva A., de Brito J. (2020). Computational models applied to the service life prediction of External Thermal Insulation Composite Systems (ETICS). J. Build. Eng..

[18] Landolfi R., Nicolella M. (2022). Durability Assessment of ETICS: Comparative Evaluation of Different Insulating Materials. Sustainability.

[19] Gonçalves M., Simões N., Serra C., Almeida J., Flores-Colen I., Vieira de Castro N., Duarte L. (2021). Onsite monitoring of ETICS comparing different exposure conditions and insulation materials. J. Build. Eng..

[20] Kvande T., Bakken N., Bergheim E., Thue J. (2018). Durability of ETICS with Rendering in Norway—Experimental and Field Investigations. Buildings.

[21] Parracha J.L., Borsoi G., Flores-Colen I., Veiga R., Nunes L., Dionísio A., Gomes M.G., Faria P. (2021). Performance parameters of ETICS: Correlating water resistance, bio-susceptibility and surface properties. Constr. Build. Mater..

[22] Xu H., Wang H., Huo Q., Qin Y., Zhou H. (2023). Comparative study of Chinese, European and ISO external thermal insulation composite system (ETICS) standards and technical recommendations. J. Build. Eng..

[23] Parracha J.L., Veiga R., Flores-Colen I., Nunes L. (2023). Toward the Sustainable and Efficient Use of External Thermal Insulation Composite Systems (ETICS): A Comprehensive Review of Anomalies, Performance Parameters, Requirements and Durability. Buildings.

[24] Norvaišienė R., Buhagiar V., Burlingis A., Miškinis K. (2020). Investigation of mechanical resistance of external thermal insulation composite systems (ETICS). J. Build. Eng..

[25] Xiong H., Yuan K., Wen M., Yu A., Xu J. (2019). Influence of pore structure on the moisture transport property of external thermal insulation composite system as studied by NMR. Constr. Build. Mater..

[26] Garecki M., Bednarczyk K., Świercz P., Pogorzelec P., Kulesza M., Michałowski B. (2022). Dylatacja 25-go metra. Skutki—Przyczyny—Realne Wyzwania, Proceedings of the International Conference ETICS, Łochów, Poland, 11–13 May 2022.

[27] Norvaišienė R., Krause P., Buhagiar V., Burlingis A. (2021). Resistance of ETICS with Fire Barriers to Cyclic Hygrothermal Impact. Sustainability.

[28] Amaro B., Saraiva D., De Brito J., Flores-Colen I. (2013). Inspection and diagnosis system of ETICS on walls. Constr. Build. Mater..

[29] Asphaug S.K., Time B., Kvande T. (2021). Moisture Accumulation in Building Façades Exposed to Accelerated Artificial Climatic Ageing—A Complementary Analysis to NT Build 495. Buildings.

[30] Freitas S., de Freitas V.P. (2016). Cracks on ETICS along thermal insulation joints: Case study and a pathology catalogue. Struct. Surv..

[31] European Organization for Technical Assesment (2013). Guideline for European Technical Approval ETAG 004 External Thermal Insulation Composite Systems (ETICS).

[32] Krause P., Norvaišienė R. (2020). Temperature and humidity tests of the ETICS system with a fire barrier. Mater. Bud..

[33] Maia J., Ramos N.M.M., Veiga R. (2019). A new durability assessment methodology of thermal mortars applied in multilayer rendering systems. Constr. Build. Mater..

[34] European Severe Weather Database. https://eswd.eu/.

[35] Bochen J. (2015). Weathering effects on physical–chemical properties of external plaster mortars exposed to different environments. Constr. Build. Mater..

[36] Ślusarek J., Orlik-Kożdoń B., Bochen J., Muzyczuk T. (2020). Impact of the imperfection of thermal insulation on structural changes of thin-layer façade claddings in ETICS. J. Build. Eng..

[37] Griciutė G., Bliūdžius R. (2015). Study on the microstructure and water absorption rate changes of exterior thin-layer polymer renders during natural and artificial ageing. Medziagotyra.

[38] (2015). Thermal Insulation Products for Buildings—Factory Made Expanded Polystyrene (EPS) Products—Specificatio.

[39] Sulakatko V., Vogdt F. (2018). Construction Process Technical Impact Factors on Degradation of the External Thermal Insulation Composite System. Sustainability.

[40] Piekarczuk A., Sudoł E., Mazurek A. (2024). Measurement analysis of Large-Area elements of external thermal insulation Composite Systems using 3D scanning techniques. Meas. J. Int. Meas. Confed..

[41] (2018). Paints and Varnishes Determination of Water-Vapour Transmission Properties. Cup Method.

[42] (1988). Vertical Building Elements. Impact Resistance Tests. Impact Bodies and General Test Procedures.

[43] Bochen J. (2009). Study on the microstructure of thin-layer facade plasters of thermal insulating system during artificial weathering. Constr. Build. Mater..

[44] Uygunoğlu T., Özgüven S., Çalış M. (2016). Effect of plaster thickness on performance of external thermal insulation cladding systems (ETICS) in buildings. Constr. Build. Mater..

[45] Malanho S., Veiga M. (2020). do R. Bond strength between layers of ETICS—Influence of the characteristics of mortars and insulation materials. J. Build. Eng..

[46] Yuan K., Xiong H., Wen M., Xu J. (2022). Visualization of localized deformation of external thermal insulation composite systems during aging. Appl. Therm. Eng..

[47] Sudoł E., Dębski D., Zamorowska R., Francke B. (2018). Impact resistance of external thermal insulation systems. MATEC Web Conf..

